# First-year emotional health after liver transplantation: a prospective cohort study

**DOI:** 10.3389/fpsyg.2026.1815413

**Published:** 2026-05-19

**Authors:** Victor Fernandez-Alonso, Ana Maria Hernandez-Matias, Raquel Gonzalez-Hervias, Ana Fernandez-Alonso, Manuela Perez-Gomez, Maria Nieves Moro-Tejedor

**Affiliations:** 1 Escuela Universitaria de Enfermería Cruz Roja-UAM, Madrid, Spain; 2 Instituto de Investigacion Sanitaria Gregorio Maranon, Madrid, Spain; 3 Hospital General Universitario Gregorio Maranon, Madrid, Spain; 4 La Fundacion para la Investigacion Biomedica del Hospital Gregorio Maranon, Madrid, Spain

**Keywords:** anxiety, depression, liver transplantation, medication adherence, mental health

## Abstract

Liver transplantation profoundly influences a patient’s health and quality of life, closely tied to their ability to adapt to post-transplant. This study analyzed anxiety, depression, and emotional-behavioral changes during the first-year post-transplant through an exploratory cohort study conducted from December 2019 to February 2022. Data were collected on sociodemographic and clinical factors, the Transplant Effects Questionnaire, and anxiety/depression variables. Seventy-six patients were enrolled (mean age: 55.96 years, 69.7% male). Sixty-eight participants completed all study time points and were included in the repeated-measures longitudinal analyses. Findings showed a significant reduction in worry and responsibility over time, with minor improvements in disclosure. Guilt and adherence remained stable. Anxiety and depression were correlated with worry and disclosure, affecting mental health during the recovery period. The transplantation process is complex and dynamic, highlighting the importance of promoting adherence to immunosuppression treatment for long-term survival. Addressing anxiety and depression through tailored interventions that encourage better care practices, reduce guilt, and enhance disclosure can significantly improve adherence and mental health outcomes. Nurses play a critical role in implementing holistic interventions, fostering patient well-being, and supporting their life post-transplant.

## Introduction

Liver transplantation (LT) is the most effective treatment for all types of liver failure (Millson et al., 2020). According to the latest European data, the number of LT performed in 2023 was 10,684 (European Directorate for the Quality of Medicines & HealthCare, 2024). In Spain, the National Transplant Organization (ONT) reported that 1,344 LT were performed in 2024, indicating a rate of 27.6 per million population (Organización Nacional de Trasplantes, Ministerio de Sanidad, 2025). The average age of patients undergoing LT was 56 years old, with 52% of cases attributable to cirrhosis (Organización Nacional de Trasplantes, Ministerio de Sanidad, 2025).

LT is a complex, life-altering process that affects not only the physical health of patients but also their emotional and psychological stability. Patients in the terminal phase of liver disease face limitations in their lives (Peng et al., 2019). LT recipients are at high risk of psychological distress. Social, psychological, and psychiatric factors appear to influence both morbidity and mortality before and after the transplant (Golfieri et al., 2019). Depression is one of the most extensively studied psychological conditions in the field of organ transplantation. Other symptoms observed throughout the process include stress related to living with a life-threatening illness, reliving negative experiences associated with the disease, avoidance of stress-inducing factors, and feelings of excitement and anticipation regarding the arrival of the organ (Annema et al., 2017). Additionally, LT recipients may develop a sense of responsibility toward the donor, their family, and the medical team (Golfieri et al., 2019; Annema et al., 2017). Understanding the individual emotional and psychological journey of each patient is essential to prevent psychological morbidity and to promote post-traumatic growth, effective emotional regulation, and proper self-care. These aspects are crucial for improving the overall wellbeing and long-term outcomes of LT recipients (Saiyu et al., 2023; Biyyala et al., 2023). Noteworthy improvements in survival rates, along with improvements in survival, mental health, and quality of life have been documented after LT (Yang et al., 2014; Albayrak et al., 2023). Nevertheless, there is a paucity of research addressing the emotional needs of these patients and the most effective methods to provide them with support. After a successful LT, patients have to adapt to life on a lifelong regimen of immunosuppressive medications and lifestyle rules, but they may also have to deal with serious, potentially life-threatening complications such as graft rejection or development of cardiovascular diseases or cancer (Annema et al., 2017). Quality of life after liver transplantation is influenced by multiple factors (e.g., clinical course, comorbidities, social support, and psychological well-being), and patients’ coping and adaptation processes represent key modifiable contributors within this broader framework (Pérez-San-Gregorio et al., 2017). Emotions and care behaviors are complex phenomena that encompass a wide array of cognitive, emotional and behavioral dimensions subsequent to organ transplantation (Bergamos Trevizan et al., 2017). The term psychological fragility describes a person’s ability to remain resilient in the face of stressors in terms of both mood and cognition (Bajaba et al., 2024). For transplant recipients, resilience manifests in their ability to cope with the psychological burden of receiving an organ, navigate complex emotions such as guilt toward the donor, and maintain consistent adherence to immunosuppressive treatment despite potential side effects or lifestyle disruptions. The use of standardized psychometric instruments, such as the Transplant Effects Questionnaire (TxEQ) enables the evaluation and comparison of these effects (Pérez-San-Gregorio et al., 2018; Ziegelmann et al., 2002).

Acknowledging the patient’s perspective on their health is commonly practiced when assessing the benefits and risks of therapeutic interventions. Patient-reported outcomes (PRO) serve as a cornerstone in regulatory decision-making processes. These measures provide indispensable insights into treatment effects, the balance between benefits and risks, and the substantiation of therapeutic claims. Such data enables regulatory authorities to ensure that medical interventions are assessed not solely for their clinical efficacy but also for their influence on patients’ quality of life and overall well-being, thereby rendering the approval process more patient-focused. The integration of PRO into regulatory frameworks aligns with the evolving emphasis on patient-centered healthcare and the incorporation of real-world evidence into the evaluation of medical treatments (Minvielle et al., 2022; U.S Food and Drugs, 2024; European Medicine Agency, 2022). To address this gap, we prospectively evaluated emotional and behavioral responses captured by TxEQ-Spanish and their relationship with anxiety and depressive symptoms (HADS) at 1, 6, and 12 months after liver transplantation in a real-world Spanish transplant follow-up program. The primary aim of this study was to assess the relationships between worry, guilt, disclosure, responsibility, and adherence with anxiety, and depressive symptoms in a cohort of LT patients from a cadaveric donor during the initial year after LT.

## Methods

### Study design

We conducted an exploratory observational study using a longitudinal cohort design, which included both retrospective and prospective phases.

### Setting and sampling

The present study was carried out in the LT unit of the Gregorio Marañón General University Hospital, a tertiary hospital in Madrid, Spain. This hospital center exclusively performs adult LT, with an average of 42.25 LTs performed per year from 2018 to 2021 (Organización Nacional de Trasplantes, Ministerio de Sanidad, 2023). The study population comprised all adult patients who underwent LT from cadaveric donors.

### Participants/study size

The study population comprised all adult patients on the waiting list for their first elective LT at the LT unit of the Gregorio Marañón General University Hospital in Madrid, Spain, between December 2019 and February 2022. Patients were selected once they were accepted by the LT committee as candidates. Eligible patients received verbal and written information during the transplant evaluation visit. To minimize undue influence, participation was presented as independent from clinical decision-making and routine care. Written informed consent was obtained after patients had sufficient time to consider participation. Participation was voluntary, refusal did not affect clinical care, and participants could withdraw at any time without consequences. Research consent was separate from clinical consent and did not influence listing decisions or clinical management.

### Inclusion and exclusion criteria

All patients who received LT during the designated study period were included. Patients who had previously received another organ transplant were excluded. Patients who required re-transplantation more than 1 month after the index LT were withdrawn from follow-up to minimize clinical heterogeneity and because their subsequent recovery and psychosocial trajectory may substantially differ from standard post-LT follow-up (Cuevas López et al., 2020). Patients who did not complete data collection for the duration of the study were also withdrawn from follow-up.

### Variables

Sociodemographic variables were collected: age, in years; sex: male/female; marital status: single, in a relationship-married, separated/divorced or widowed; educational level: primary, secondary, high school, vocational training or university; employment status: not working-unemployed, active or retired-pensioner; and religion: not religious, practicing catholic, non-practicing catholic, Islamic, and other religions (Paglione et al., 2019). Clinical variables associated with cardiovascular risk factors were also collected: obesity, diabetes mellitus (DM), high blood pressure, dyslipidemia and smoking. Variables related to liver disease were collected at the time of inclusion on the waiting list: prognosis of the patient with cirrhosis and etiology, determined through functional analytical tests, and liver serologies. The times, in days, on the waiting list, hospital admission after LT. The alcohol consumption variable was not collected because to be a candidate for a liver transplant, abstinence for a period of 6 months or more is necessary. By including these variables, we aimed to provide a more comprehensive understanding of the factors influencing mental health outcomes in LT patients, and to ensure that our analyses could account for relevant confounders or moderators.

### Data sources/measurement

Data collection started after Ethics Committee approval had been obtained and participants had provided written informed consent. The study included a retrospective and a prospective phase. First, clinical and baseline variables were retrieved retrospectively from the electronic health record (EHR) for patients transplanted between January 2020 and February 2021. Subsequently, patients were screened and enrolled prospectively by the liver transplant (LT) outpatient clinic nurse. Clinical data (e.g., liver disease characteristics, cardiovascular risk factors, waiting-list time, length of stay, readmissions, and transplant-related outcomes) were collected via EHR chart review by the research team. Patient-reported outcomes (TxEQ-Spanish and HADS) were collected at 1, 6, and 12 months post-LT. Survey administration and follow-up assessments were scheduled to coincide with patients’ routine post-transplant outpatient visits. During scheduled outpatient clinic visits, participants completed the self-administered questionnaires in a private setting and returned them to the LT nurse before leaving the clinic.

### Instrument with validity and reliability

#### Hospital anxiety and depression scale

The Hospital Anxiety and Depression Scale (HADS) is a PRO measure developed to identify depression and anxiety in patients in non-psychiatric wards (Rothmund et al., 2023). The HADS has been designed specifically for use in patients with somatic illnesses in non-psychiatric hospital settings and therefore excludes somatic symptoms and focuses on the affective aspects of depression and anxiety. The HADS consists of 14 items that form two 7-item scales for depression (HADD) and anxiety (HADA), respectively. Anxiety is more behaviorally oriented and involves physiological and emotional responses such as restlessness, tension, and heightened alertness. They are rated on a 4-point scale. Scores for each subscale can vary from 0 to 2. Values between 11 and 21 are considered probable cases of anxiety and/or depression. Internal consistency, evaluated using Cronbach’s alpha, was 0.90 for the complete scale, 0.84 for the depression subscale and 0.85 for the anxiety subscale (Herrero et al., 2003).

#### Transplant effects questionnaire (TxEQ) Spanish

The Transplant Effects Questionnaire (TxEQ) is a questionnaire of five emotional and behavioral subscales about the transplant that evaluate from feelings of guilt towards the donor, to responsibility, compliance with immunosuppressive treatment, worry, and disclosure. Worry tends to be a cognitively driven process, often involving repetitive thoughts about potential negative outcomes. Disclosure subscale specifically evaluates the extent to which transplant recipients feel comfortable sharing their transplant experience with others. It captures attitudes toward openness, perceived stigma, and the degree to which the transplant is integrated into the individual’s personal identity. Higher scores on this subscale indicate greater ease in discussing the transplant with others, which has been associated with better psychological adjustment and social support. These dimensions are deeply intertwined with the concept of resilience, which refers to an individual’s capacity to adapt positively in the face of adversity, stress, or trauma. The TxEQ, by capturing these emotional and behavioral responses, provides valuable insight into how resilient a patient is in managing the challenges of post-transplant life. In this way, the questionnaire not only evaluates psychological adjustment but also indirectly reflects the strength and flexibility of the recipient’s resilience. The Spanish version includes 22 items rated on a 5-point Likert scale ranging from “strongly agree” to “strongly disagree,” with a maximum score of 5 and a minimum score of 1. The score for each subscale is the average of the scores of the elements that comprise it. All dimensions of the TxEQ-Spanish met internal consistency and Cronbach’s alpha coefficients ranged between 0.77 and 0.91 (Pérez-San-Gregorio et al., 2018).

### Bias

To minimize bias, retrospective data were extracted by the same researcher from the electronic medical record. Questionnaires were administered and collected systematically by the same nurse, reducing observer and interpretation bias.

### Quantitative variables/ data analysis

An exploratory analysis was conducted to identify potential outliers and extreme values using graphical inspection (boxplots and histograms) and distributional checks at each measurement occasion (1, 6, and 12 months post-LT). Because data were collected longitudinally, analyses were performed using repeated measures across these measurement occasions rather than defining case groups. These case groups were analyzed to assess the evolution of emotional and behavioral effects and their correlation with mental health outcomes. Sociodemographic variables were recoded to facilitate analysis while preserving relevant information.

Normality was assessed using the Kolmogorov–Smirnov test and complemented by visual inspection of histograms and Q–Q plots. The relationship between the dimensions of the TxEQ-Spanish and HADS was evaluated using Pearson’s correlation coefficient for normally distributed variables. Spearman’s rho correlation coefficient was used for the HADA and HADD subscales because these variables did not meet normality assumptions. Correlation strength was interpreted as follows: ≤ 0.29 = weak; 0.30–0.49 = low; 0.50–0.69 = moderate; and > 0.70 = strong. Statistical significance was established at *α* < 0.05 (Rothmund et al., 2023). HADS, which did not meet the assumptions of normality and showed high variability, were analyzed using the Friedman test to compare the mean scores at the three time points. Changes in TxEQ-Spanish dimensions over time were analyzed using a linear mixed-effects regression model. This model accounted for repeated measures within individuals by including patients as random effects and TxEQ-Spanish dimensions as fixed effects. An unstructured covariance matrix was used to allow flexibility in modeling correlations between repeated measures. Due to the exploratory nature of the study, multivariable analyses were not performed. Statistical analyses focused on descriptive statistics, bivariate associations, and linear mixed-effects models to explore patterns in the data (Krueger and Tian, 2004). All analyses were performed using STATA version 16.

### Ethical considerations

This study, and its written consent, was approved by the Gregorio Marañón General University Hospital Ethics Committee (code IMPACT_TH of the 02/2021 min) and was carried out in accordance with the principles articulated in the Declaration of Helsinki (World Medical Association, 2013), as well as Regulation (EU) 2016/679 of the European Parliament and of the Council of April 27, 2016 on Data Protection (European Parliament, 2016) ensuring correct data coding and pairing. To ensure voluntary and informed consent, participants are provided with comprehensive information regarding the study’s purpose, procedures, potential risks, and benefits before agreeing to take part. This process includes a written consent form detailing these aspects, which must be signed by each participant after they have had the opportunity to ask questions and receive clarifications. Confidentiality is maintained through strict data protection protocols, including secure storage, restricted access, and the use of anonymized identifiers to prevent unauthorized disclosure of sensitive information. Personal data were handled in accordance with applicable ethical and legal standards to safeguard participant privacy. To protect anonymity, identifying details are either removed or replaced with coded identifiers in all records, publications, and shared datasets. Researchers ensure that findings cannot be traced back to specific individuals, thereby maintaining privacy and ethical integrity throughout the study. These measures collectively uphold ethical research standards and foster trust between researchers and participants.

## Results

**Figure 1:**
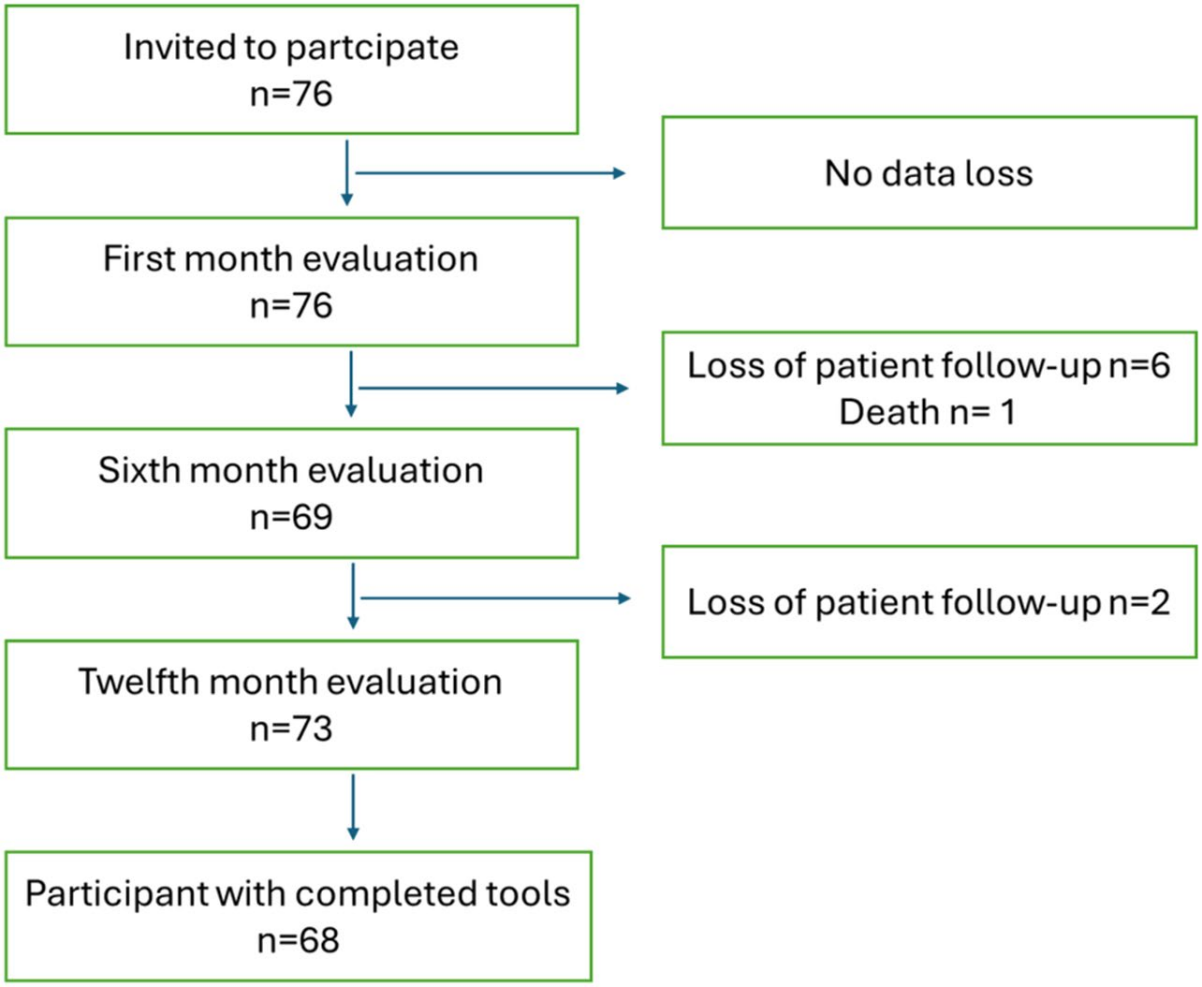
Flow chart study sample.

A total of 76 LT patients completed the 1-month assessment; 68 completed all follow-up assessments (1, 6, and 12 months) and constituted the repeated-measures cohort for longitudinal TxEQ analyses. All participants completed the 1-month assessment; 9.21% were missing at 6 months and 2.66% at 12 months (Figure 1). The patients’ mean age was 55.96 years (SD = 11.19). At the time of waiting list inclusion, 48.7% (*n* = 37) were ≥ 60 years old. Regarding liver disease, 39.5% (*n* = 30) had alcohol-related liver disease. The remaining social and clinical variables are described in Supplementary Table 1. For those patients included who were candidates for elective transplantation, the median number of days on the waiting list was 114.50 [IQR 202.25–58.75], and the median number of days from transplantation to the patient’s discharge home was 17 [IQR 23–13]. Only 7.9% (*n* = 6) needed immediate re-transplantation due to 50% arterial thrombosis and 50% primary graft malfunction. The one-year survival rate following liver transplantation was 98.68% (*n* = 75). There was one fatality caused by a stroke.

### TxEQ-Spanish/ HADS 1 month after LT

One month after LT *n* = 76 patients completed the surveys. Worry was significantly greater in patients with a higher academic level (*p* = 0.004). Guilt was higher in those who professed a religion (*p* = 0.043). The anxiety score was directly correlated with depression, which, in turn, was directly correlated with worry, and indirectly with disclosure. Treatment adherence demonstrated a direct significant correlation with disclosure and an indirect significant correlation with guilt (Table 1).

**Table 1:** TxEQ-Spanish and HADS correlations 1 month after LT.

TxEQ-HADS (*n* = 76)		Worry	Guilt	Disclosure	Responsibility	Adherence	HADA	HADD
Worry	*r*	1	0.206	−0.225	**0.321** ^**^	0.014	0.449**	0.223
	Two-sided *p*-value		0.074	0.051	**0.005**	0.905	**<0.001**	0.052
Guilt	*r*	0.206	1	**−0.544** ^**^	0.037	**−0.381** ^**^	0.098	−0.135
	Two-sided *p*-value	0.074		**<0.001**	0.753	**0.001**	0.399	0.245
Disclosure	*r*	−0.225	**−0.544** ^**^	1	−0.028	**0.431** ^**^	−0.271*	0.044
	Two-sided *p*-value	0.051	**<0.001**		0.811	**<0.001**	**0.018**	0.709
Responsibility	*r*	**0.321** ^**^	0.037	−0.028	1	0.046	0.012	−0.035
	Two-sided *p*-value	**0.005**	0.753	0.811		0.695	0.920	0.763
Adherence	*r*	0.014	**−0.381** ^**^	**0.431** ^**^	0.046	1	−0.145	0.090
	Two-sided *p*-value	0.905	**0.001**	**<0.001**	0.695		0.212	0.438
HADA	*p*	**0.449** ^**^	0.098	**−0.271** ^**^	0.012	−0.145	1	0.374**
	Two-sided *p*-value	**<0.001**	0.399	**0.018**	0.920	0.212	.	**0.001**
HADD	*p*	0.223	−0.135	0.044	−0.035	0.090	0.374**	1
	Two-sided *p*-value	0.052	0.245	0.709	0.763	0.438	**0.001**	.

r = Pearson correlation coefficient; ρ = Spearman’s rho correlation coefficient (used for HADA and HADD); *. The correlation is significant at the 0.05 level (two-sided); **. The correlation is significant at the 0.01 level (two-sided); TxEQ-Spanish: Transplant Effects Questionnaire Spanish; HADS: Hospital Anxiety Depression Scale; LT: Liver Transplantation; HADA/HADD: Hospital Anxiety Scale/ Hospital Depression Scale. Bold values: statistically significant.

### TxEQ-Spanish/HADS 6 months after LT

Six months after LT *n* = 69 patients completed the surveys. The mean worry score was found to be significantly greater among women (*p* = 0.017). The guilt scores were greater in those who professed a religion (*p* = 0.023). The HADS scores exhibited a direct correlation with worry, and anxiety exhibited an indirect correlation with disclosure. Notably, adherence to treatment demonstrated consistent correlations with the initial month post-LT. Those patients with greater worry had greater responsibility and anxiety/depression, while demonstrating a reduced propensity for disclosing their status as transplant recipients (Table 2).

**Table 2:** TxEQ-Spanish and HADS correlations to the sixth of the TH.

TxEQ-HADS (*n* = 69)		Worry	Guilt	Disclosure	Responsibility	Adherence	HADA	HADD
Worry	*r*	1	0.188	**−0.254** ^*^	**0.248** ^*^	−0.049	0.509**	0.361**
	Two-sided *p*-value		0.122	**0.036**	**0.040**	0.687	**<0.001**	**0.002**
Guilt	*r*	0.188	1	**−0.366** ^**^	0.162	**−0.319** ^**^	0.019	−0.051
	Two-sided *p*-value	0.122		**0.002**	0.184	**0.008**	0.879	0.674
Disclosure	*r*	**−0.254** ^*^	**−0.366** ^**^	1	−0.046	**0.491** ^**^	−0.248*	−0.193
	Two-sided *p*-value	**0.036**	**0.002**		0.710	**<0.001**	**0.040**	0.112
Responsibility	*r*	**0.248** ^*^	0.162	−0.046	1	−0.020	−0.061	−0.184
	Two-sided *p*-value	**0.040**	0.184	0.710		0.867	0.617	0.131
Adherence	*r*	−0.049	**−0.319** ^**^	**0.491** ^**^	−0.020	1	−0.084	0.057
	Two-sided *p*-value	0.687	**0.008**	**<0.001**	0.867		0.493	0.641
HADA	*p*	**0.509** ^**^	0.019	**−0.248** ^*^	−0.061	−0.084	1	**0.521** ^**^
	Two-sided *p*-value	**<0.001**	0.879	**0.040**	0.617	0.493	.	**<0.001**
HADD	*p*	**0.361** ^**^	−0.051	−0.193	−0.184	0.057	**0.521** ^**^	1
	Two-sided *p*-value	**0.002**	0.674	0.112	0.131	0.641	**<0.001**	

r = Pearson correlation coefficient; ρ = Spearman’s rho correlation coefficient (used for HADA and HADD); * The correlation is significant at the 0.05 level (two-sided); **. The correlation is significant at the 0.01 level (two-sided); TxEQ-Spanish: Transplant Effects Questionnaire Spanish; HADS: Hospital Anxiety Depression Scale; LT: Liver Transplantation; HADA/HADD: Hospital Anxiety Scale/ Hospital Depression Scale; TxEQ-Spanish: Transplant Effects Questionnaire Spanish. Bold values: statistically significant.

### TxEQ-Spanish/ HADS 12 months after LT

One year after LT *n* = 73 patients completed the surveys. Levels of worry were significantly greater in those who professed a religion (*p* = 0.037). The anxiety score increased its direct correlations worry, and indirect correlations with disclosure. Anxiety and depression maintained a direct correlation throughout the first year after LT. Adherence to treatment maintained its respective correlations in the first- and sixth months after LT directly with guilt, and indirectly with disclosure. Disclosure of the situation as a transplant patient was directly correlated with adherence and indirectly with guilt, worry, and anxiety (Table 3).

**Table 3:** TxEQ-Spanish and HADS correlations at the twelfth of TH.

TxEQ-HADS (*n* = 73)		Worry	Guilt	Disclosure	Responsibility	Adherence	HADA	HADD
Worry	*r*	1	**0.258** ^*^	**−0.350** ^**^	0.197	−0.199	**0.570** ^**^	**0.509** ^**^
	Two-sided *p*-value		**0.027**	**0.002**	0.095	0.091	**<0.001**	**<0.001**
Guilt	*r*	**0.258** ^*^	1	**−0.320** ^**^	**0.291** ^*^	**−0.301** ^**^	0.206	0.083
	Two-sided *p*-value	**0.027**		**0.006**	**0.012**	**0.010**	0.080	0.486
Disclosure	*r*	**−0.350** ^**^	**−0.320** ^**^	1	−0.033	**0.480** ^**^	**−0.267** ^*^	−0.212
	Two-sided *p*-value	**0.002**	**0.006**		0.779	**<0.001**	**0.023**	0.071
Responsibility	*r*	0.197	**0.291** ^*^	−0.033	1	0.060	0.165	−0.047
	Two-sided *p*-value	0.095	**0.012**	0.779		0.613	0.162	0.694
Adherence	*r*	−0.199	**−0.301** ^**^	**0.480** ^**^	0.060	1	−0.196	−0.141
	Two-sided *p*-value	0.091	**0.010**	**<0.001**	0.613		0.096	0.236
HADA	*p*	**0.570** ^**^	0.206	**−0.267** ^*^	0.165	−0.196	1	**0.599** ^**^
	Two-sided *p*-value	**<0.001**	0.080	**0.023**	0.162	0.096	.	**<0.001**
HADD	*p*	**0.509** ^**^	0.083	−0.212	−0.047	−0.141	**0.599** ^**^	1
	Two-sided *p*-value	**<0.001**	0.486	0.071	0.694	0.236	**<0.001**	.

r = Pearson correlation coefficient; ρ = Spearman’s rho correlation coefficient (used for HADA and HADD); *. The correlation is significant at the 0.05 level (two-sided); **. The correlation is significant at the 0.01 level (two-sided); TxEQ-Spanish: Transplant Effects Questionnaire Spanish; HADS: Hospital Anxiety Depression Scale; LT: Liver Transplantation; HADA/HADD: Hospital Anxiety Scale/ Hospital Depression Scale; TxEQ-Spanish: Transplant Effects Questionnaire Spanish. Bold values: statistically significant.

### Analysis of differences in TxEQ during the first year post-LT

**Figure 2:**
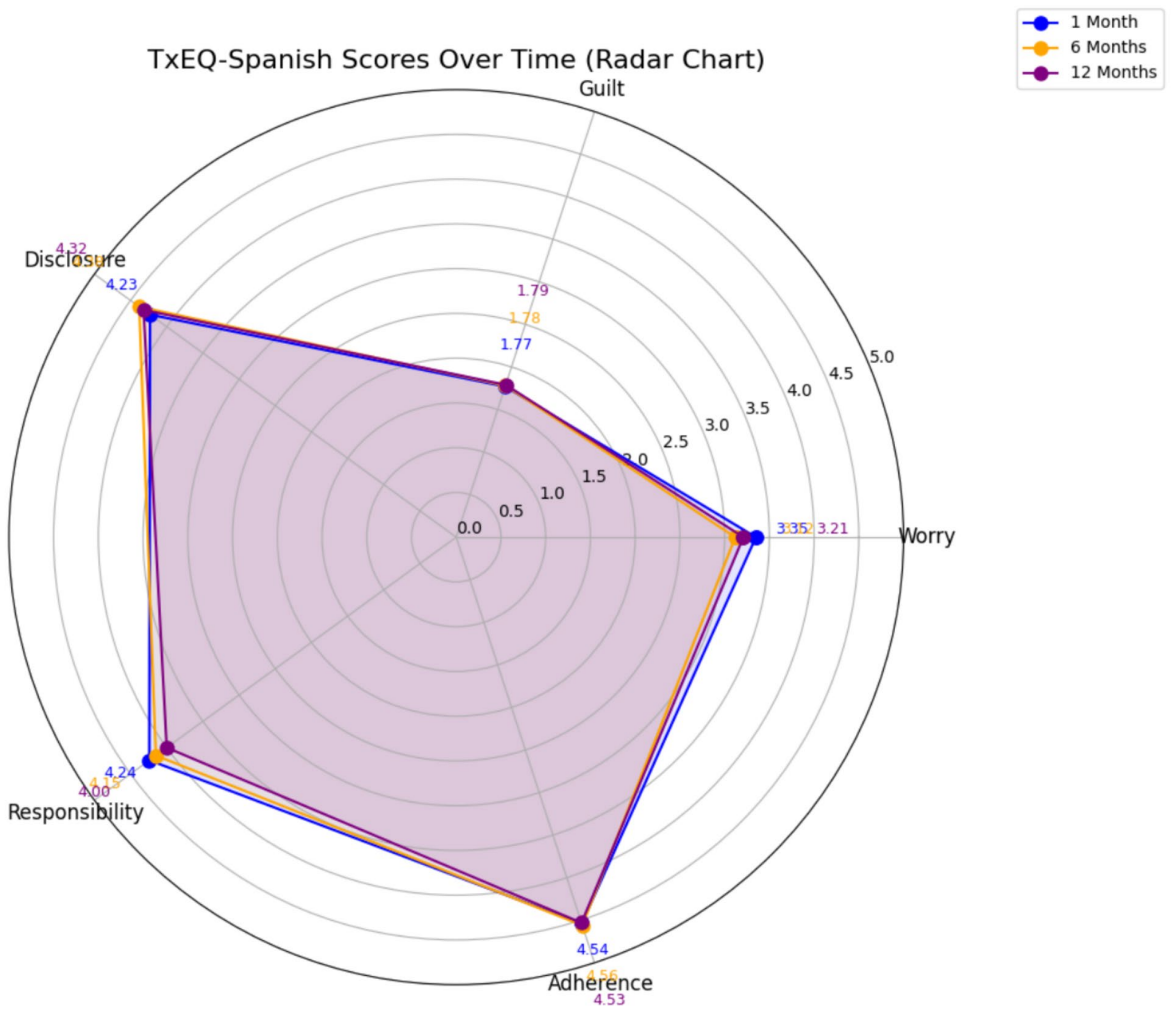
TxEQ-Spanish mean scores across three timepoints during the first year after transplantation.

**Figure 3:**
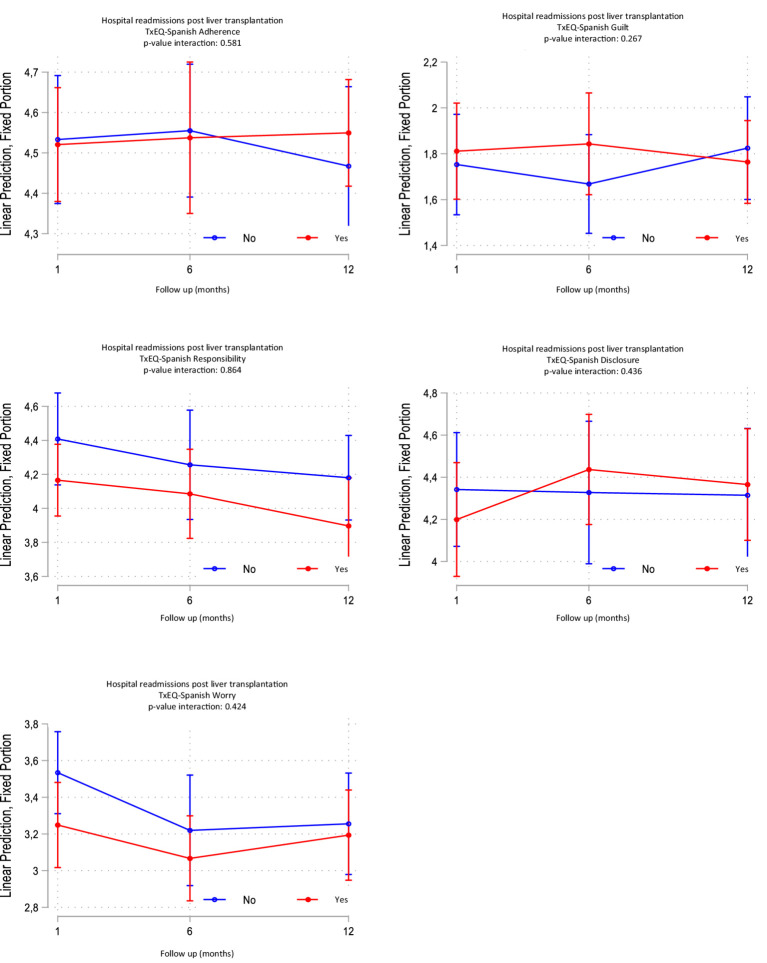
Interaction of hospital readmissions on emotional health after liver transplantation.

A total of *n* = 68 patients completed the study surveys. Results show key trends in emotional and behavioral dimensions among liver transplant patients over time. Figure 2 shows the evolution of the five dimensions of the TxEQ-Spanish at 1, 6, and 12 months after LT. The findings described statistically significant differences between worry score means with a small effect size. The decreasing difference in mean scores of worry between the first and sixth month after transplantation was statistically significant *p* = 0.016 [95% CI: 0.34–0.427], reflecting improved adjustment over time. The responsibility scores also showed a significant decrease with a small effect size. Our findings describe a decreasing difference in mean scores from 1 month to 12 months which was statistically significant *p* = 0.018 [95% CI: 0.033–0.452]. No statistically significant differences were observed in the disclosure score, guilt, or treatment adherence (Table 4). These variables remained stable during the first year, suggesting a gradual adjustment to the demands of post-transplant care. Disclosure scores increased slightly, although without reaching statistical significance. Guilt and adherence scores remained stable across time points, with no significant differences observed (Supplementary Table 2). These patterns suggest strong adherence to treatment and disclosure behaviors, with small fluctuations in emotional dimensions. The interactions between adjustment variables (age, sex, etiology, and time since transplantation) and TxEQ-Spanish dimensions were evaluated at different post-hospitalization follow-up points. Regarding the impact of clinical course, the interaction between hospital readmission and time did not reach statistical significance for any of the TxEQ dimensions (all *p* > 0.05), indicating that the evolution of these psychological variables remained consistent regardless of whether patients required re-hospitalization. Specifically, Adherence (*p* = 0.581), Guilt (*p* = 0.267), Responsibility (*p* = 0.864), Disclosure (*p* = 0.436), and Worry (*p* = 0.424) showed stable patterns over the first year (Table 5). Although Worry showed a marginal trend at the 1-month mark (*p* = 0.084) in re-admitted patients, this difference dissipated by the end of the 12-month follow-up. These findings suggest relative stability in the evaluated dynamics, with adjustment variables such as age, sex, and etiology showing no significant influence on the TxEQ-Spanish dimensions over time (Figure 3).

**Table 4:** Estimated differences in TxEQ in liver transplant patients at 1 month, 6 months and 12 months after LT.

TxEQ-Spanish **(*n* = 68)**	1 month x̅ (SD)	6 month x̅ (SD)	12 month x̅ (SD)	*F*	***p*-value**	η^**2**^
Worry	3.35 (0.82)	3.12 (0.86)	3.21 (0.86)	3.586	0.030*	0.051
Guilt	1.77 (0.69)	1.78 (0.72)	1.79 (0.66)	0.046	0.955	0.001
Disclosure	4.23 (0.89)	4.38 (0.92)	4.32 (0.91)	0.916	0.393	0.013
Responsibility	4.24 (0.80)	4.15 (0.91)	4 (0.89)	3.566	0.031*	0.051
Adherence	4.54 (0.46)	4.56 (0.57)	4.53 (0.46)	0.135	0.856	0.002

*p < 0.05; x̅: Mean; SD: Standard deviation. Bold values: statistically significant.

**Table 5:** Linear Mixed-effects Model TxEQ-Spanish scores adjusted by age, sex, liver disease etiology, and the waiting list duration.

TxEQ-Spanish dimension	*T* = 1 (p)	*T* = 6 (p)	*T* = 12 (p)	Interaction (p)
Adherence	0.913	0.890	0.510	0.581
Guilt	0.706	0.260	0.669	0.267
Responsibility	0.157	0.413	0.130	0.864
Disclosure	0.468	0.614	0.808	0.436
Worry	0.084	0.433	0.746	0.424

T = time (month after liver transplant).

Anxiety and depression scores measured using the HADS scale at 1, 6, and 12 months after LT showed moderate variability between the different time points. For anxiety, the median score was 5.00 at 1 month [IQR = 2.00–7.00], 4.00 at 6 months [IQR = 1.00–7.00] and 5.00 at 12 months [IQR = 2.00–8.00]. For depression, the medians were 3.00 at 1 month [IQR = 1.00–4.00], 1.50 at 6 months [IQR = 1.00–3.00] and 2.00 at 12 months [IQR = 1.00–5.00]. These distributions indicate that most patients scored in the lower range of the scale, with some variability over time. Non-parametric analysis using the Friedman test showed no statistically significant differences in anxiety between the different time points [χ^2^ (2) = 5.03, *p* = 0.081], suggesting stability in anxiety levels during the first year after transplantation. However, depression scores showed a statistically significant difference [χ^2^ (2) = 7.24, *p* = 0.027], with mean ranges indicating a slight decrease at 6 months (median = 1.50, IQR = 1.00–3.00) followed by an increase at 12 months (median = 2.00, IQR = 1.00–5.00). These findings may reflect a transient improvement in depressive symptoms in the middle of the year, with a tendency to increase at the end of the first year.

Correlation analyses revealed relationships between TxEQ-Spanish dimensions and mental health outcomes. Throughout the study, worry showed a moderate direct correlation with anxiety. This overlap may reflect the transition from a cognitive state (worry) to a more activated emotional and behavioral state (anxiety), highlighting the importance of understanding their distinct yet interconnected roles in psychological assessment. Guilt showed a low indirect correlation was significantly associated with both anxiety and adherence. Disclosure showed a weak indirect correlation with anxiety, and low direct correlation with adherence to treatment. These findings underscore the interconnected nature of emotional and behavioral effects and mental health in LT patients.

## Discussion

This study provides longitudinal evidence from the first year after LT on TxEQ emotional-behavioral domains and their associations with anxiety and depressive symptoms, based on repeated assessments conducted during routine clinical follow-up. By offering context-specific real-world data, our findings help to characterize the dynamic emotional trajectories observed during early recovery and may inform psychosocial screening and nursing interventions in post-LT care. The emotional and behavioral responses experienced during the initial year following LT are complex and multifactorial. As time elapsed, there was a decline in worry and responsibility. Guilt and adherence to treatment remained constant during the first year, while disclosure of the new status as a transplant patient improved slightly. These effects correlated with anxiety and depression, impacting the patient’s emotional and mental health.

The absence of strong associations between sociodemographic or clinical variables and emotional outcomes following liver transplantation suggests that psychological adaptation may be primarily driven by individual coping mechanisms. This finding supports the integration of personalized psychosocial support into routine clinical care to promote emotional wellbeing and adherence during recovery. Although some studies indicate that sociodemographic factors (e.g., age, sex, education) and clinical variables (e.g., time since transplantation, etiology of liver disease) may show limited associations with psychological outcomes, these relationships are generally weak or inconsistent. In contrast, individual psychological factors—such as coping style, resilience, and active adaptation—are more strongly linked to emotional well-being and quality of life after liver transplantation (Lieber et al., 2023; Bardak et al., 2025; Nickel et al., 2002). Furthermore, ineffective coping and depressive symptoms have been described as stronger predictors of poor mental health outcomes than clinical or demographic variables (Nickel et al., 2002).

Our findings suggest that the emotional-behavioral dimensions of the TxEQ-Spanish appeared stable despite hospital readmissions. This stability implies that the patient’s commitment to treatment (Adherence) and their psychological burden (Guilt and Responsibility) are likely driven by internal coping mechanisms and pre-existing psychological traits rather than by the immediate clinical course of recovery. From a clinical perspective, these results are encouraging, as they suggest that even patients facing a more complex medical recovery can maintain high levels of adherence and stable emotional health, provided they receive consistent longitudinal support.

The resilience capacity of chronic patients is an aspect that must be considered, in an effort to improve the capacities to overcome and cope with the consequences of the disease (Kim et al., 2019). Studies have been conducted to examine the experiences of transplant patients, the process they undergo, and the psychological impact of the transplant (Scheel et al., 2019; Milaniak et al., 2014). The use of the Spanish version of the TxEQ has allowed a deeper understanding of this experience, including the emotions that arise during the process, as well as the psychological well-being (Pérez-San-Gregorio et al., 2018). This approach incorporates the patient’s experience and perspective (Dean et al., 2021). In the context of LT, emotional and mental health play a critical role in patient recovery and long-term outcomes. The psychological constructs examined in this study are closely linked to the emotional well-being of LT recipients. These factors may influence how patients cope with the stress of transplantation, adjust to lifestyle changes, and engage with post-transplant care. This finding aligns with the outcomes of previous studies that also reported minimal guilt in this dimension (Scheel et al., 2019; Milaniak et al., 2014; Goetzmann et al., 2008). With respect to the other dimensions, we observed high adherence throughout the year under review, high disclosure and responsibility, as well as moderate-high concern among the participants, which is in line with previous studies (Scheel et al., 2019; Milaniak et al., 2014; Goetzmann et al., 2008). The present findings, while not measuring identical dimensions, may align with other analogous studies. They suggest that when transplant patients perceive an elevated quality of life, it is due to their increased utilization of problem-solving strategies over avoidance strategies (Tang et al., 2021; Moayed et al., 2018), hence the high scores in the dimensions of disclosure, adherence and responsibility shown in our study. These data could be related to studies that suggest that transplant patients may present coping styles focused on the problem (active coping) (Bernal, 2020). The consistent application of strong coping skills and resilience were necessary to promote whole body health and wellness and ensure a high quality of life (Bourmistrova et al., 2022).

In a different direction, we observed that, 1 month after the transplant, worry and guilt levels were higher in individuals with a higher academic level, who were employed and reported practicing a religion. Furthermore, throughout the study, anxiety levels exhibited a direct correlation with worry and guilt, and an indirect correlation with adherence to treatment and disclosure. This would support what previous studies have already indicated that patients who exhibit behaviors of greater care for their health and openly express personal feelings are less likely to experience anxiety and depression, and therefore have a better psycho-emotional evolution throughout the process (Dew et al., 2015). Therefore, it would be interesting for nursing professionals who monitor patients to focus on holistic care and encourage the expression of emotions, as this could be key in preventing symptoms of anxiety. If deemed appropriate, they could also offer psychological counseling during the process (Yıldız and Çiftçi, 2025).

Regarding depression, it has been observed that there is a direct correlation between depression and worry 6 months and 1 year after the LT. This was already seen in previous studies that indicated a direct link between excessive worry and depressed mood, as well as a diminished quality of life (Biyyala et al., 2023; Scheel et al., 2019). In the sixth month we observed that depression indirectly correlates with disclosure, indicating that patients who disclose their concerns and express their experiences may benefit from improved management of anxiety and depression (Yıldız and Çiftçi, 2025). Conversely, the failure to disclose concerns could lead to an escalation in anxiety and depression and result in a perceived worse quality of life regarding their mental health. A study conducted with kidney and liver transplant patients suggested an intervention through group therapy based on psychoeducation to enhance the patients’ coping skills and alleviate their anxiety and depression (Bernal, 2020). The same study demonstrated that interventions, such as facilitating emotional expression, addressing denial, and reducing feelings of guilt, significantly improved the emotional adaptation of transplant patients (Bernal, 2020). These interventions included structured support for seeking help, providing targeted information, and offering professional advice. This comprehensive approach fostered enhanced acceptance of their transplant status and contributed to improved mental well-being (Craig et al., 2017). It also facilitated a reduction in the denial and guilt experienced by transplant patients and enabled greater acceptance and emotional adaptation through the pursuit of assistance. Information, and advice from the professionals who accompanied them (Craig et al., 2017). It is crucial that nurses detect worry and guilt in these patients and manage these emotions. The reduction of anxiety in LT patients has been linked to improving their tolerance of uncertainty, which can be effectively addressed through the use of informational approaches and participation in psychoeducational support groups. Health professionals should adapt their interventions to strengthen patients’ resilience and support faster recovery through continuous care and multidisciplinary rehabilitation (Halpin et al., 2021). This includes implementing educational strategies and promoting physical activity, which has been shown to improve both resilience and depressive symptoms (Kaim et al., 2020; Carriedo et al., 2020). Research conducted by nursing professionals has shown that reducing anxiety in LT patients is closely linked to improving their tolerance of uncertainty (Craig et al., 2017). This can be achieved through the use of informative techniques, particularly within psychoeducational group settings, as previously supported by other studies. The incorporation of work groups in the future of the unit is a potential avenue for patient education. These groups would focus on techniques of emotional ventilation and journaling, two strategies that may facilitate emotional expression through the oral and written word, potentially preventing the accumulation and escalation of patients’ concerns (Craig et al., 2017; Yıldız, 2021). The persistence of feelings such as guilt and worry, and their association with symptoms of anxiety and depression, underscores the importance of nursing interventions that incorporate active listening, emotional education, and coping strategies. Integrating these principles into clinical practice can enhance adherence to immunosuppressive treatment and support a more balanced and meaningful recovery for the patient. Our findings support this, highlighting the importance of addressing emotional health as part of comprehensive transplant care.

### Implications for research

Our study reveals the onset of anxiety and depression symptoms, marked by worry and guilt, within the first year after transplantation. Addressing these issues through targeted nursing interventions has the potential to greatly enhance patients’ mental health and overall quality of life. This underscores the need for future research to investigate the efficacy of structured programs incorporating emotional well-being strategies, such as journaling techniques and emotional ventilation exercises (Smyth et al., 2018). These strategies, which facilitate emotional expression through both oral and written communication, may help prevent the internalization of concerns and reduce emotional distress. Future studies could investigate the impact of such interventions on patient outcomes, focusing on their potential to enhance emotional expression and overall mental health.

### Strengths and limitations of the work

This study’s strengths include the use of novel scores and questionnaires that are innovative and facilitate our expansion of knowledge regarding post-transplant processes. Consequently, we are able to focus on our nursing interventions and design new lines of research. The collection and analysis of PROMs through the TxEQ-Spanish and HADS provides valuable information for health professionals and researchers on how patients perceive their experience after a liver transplant, having been used in other kidney transplant patients (Tong et al., 2022). This can influence treatment decisions, the planning of follow-up care, and the identification of areas where further support can be provided to improve the quality of life and well-being of transplant patients. It should be noted that the Liver Transplant Unit at Gregorio Marañón General University Hospital utilizes a high-resolution consultation model, which is managed by an Advanced Practice Nurse. This approach has been instrumental in mitigating the impact of LT on patients. The presence of an Advanced Practice Nurse to educate, inform and perform clinical follow-up of patients in our study has been demonstrated to reduce the risks described above. Conversely, this study is subject to several limitations, such as the fact that it was carried out in a single center with a limited sample size. Sociodemographic and clinical variables, such as smoking habits, were collected pre-transplant. They were not collected again during the study and could have been modified and influenced the emotional state of the participants. This study included patients who required urgent immediate re-transplantation during the first month following their initial liver transplant. This decision was made because these patients remained hospitalized in the liver transplant unit and had not yet been exposed to the real-life adaptation challenges of being a transplant recipient in their everyday environment. We acknowledge this as a limitation of the study, and future research should explore the impact of immediate re-transplantation on patients’ mental health. Late re-LT recipients (>1 month after the index LT) were not followed longitudinally in this study. This limits the generalizability of our findings to this clinically distinct subgroup. Future multicenter studies should specifically evaluate emotional and behavioral trajectories in re-transplant candidates and recipients to inform counseling and tailored psychosocial interventions. This study included consecutive patients seen in a consultation and the main clinical characteristics related to liver disease were collected, therefore, it cannot be ruled out that other variables could be important or act as confounding factors that explain some of our findings. One important limitation of our study is the potential for selection bias due to the exclusion of patients who did not complete the data collection. It is possible that these individuals were experiencing higher levels of emotional stress, which may have influenced their ability or willingness to participate fully. This could result in an underestimation of emotional distress in the overall population. Although we included all eligible patients during the study period, the missing data may have introduced bias and affected the generalizability of our findings. Future studies should consider strategies to minimize missing data and explore the characteristics of non-responders to better understand their impact on study outcomes. Also, a multivariate statistical study has not been carried out to determine the existence of confounding variables. In our study we have not analyzed adherence to medical treatment at the time of pre-LT, which a previous study describes as significant (Telles-Correia et al., 2012). The lack of multivariable analyses to simultaneously adjust for confounding factors is acknowledged as a limitation. This analytical approach ensures clarity in evaluating the emotional and mental health dimensions of liver transplant patients while acknowledging the limitations inherent in the study’s exploratory design. Although some differences reached statistical significance, the observed effect sizes were small (η^2^ ≈ 0.05), indicating that the magnitude of change across timepoints was modest. This suggests that the clinical relevance of these findings may be limited. Furthermore, small effect sizes combined with the fixed sample size may have reduced the ability to detect subtle but potentially meaningful differences. Future studies should consider larger samples or alternative designs to confirm these results and explore whether these small changes have practical implications for patient care.

## Conclusion

The liver transplant process is a dynamic and complex experience for the patient. Religious processing is associated with greater worry and guilt throughout the first year post-LT. The nurse must intervene in the promotion of healthy lifestyle habits in the liver transplant population, impacting not only the physical health but with a holistic perspective on the resilience and mental health of the patient. Furthermore, an evaluation of mental health status is essential after LT. The state of anxiety/depression is correlated directly to the level of worry and guilt. Also, promoting the disclosure of the new transplant status would improve mental and emotional health. The nurse can lead a holistic multidisciplinary intervention team that includes doctors, psychologists and pharmacists, to achieve optimal results (Krittanawong et al., 2022). Promoting correct adherence to immunosuppression treatment is vital for patient survival. Evaluating the level of anxiety/depression and carrying out interventions that promote better emotional and behavioral management, reduce guilt, and increase disclosure are likely to favor optimal adherence to treatment.

## Data Availability

The raw data supporting the conclusions of this article will be made available by the authors, without undue reservation.
