# Supplementary material for: First-year emotional health after liver transplantation: a prospective cohort study
Source: Front Psychol. 2026 May 19;17:1815413. doi: 10.3389/fpsyg.2026.1815413 (PMC13226472; doi:10.3389/fpsyg.2026.1815413)
Supplement: Supplementary file 1 [file Table_1.docx]

**Supplementary Table 1. Sociodemographic and clinical variables**

| **Variables pre-Liver Transplant (n=76)** | **n** | **%** |
| --- | --- | --- |
| **Gender** |  |  |
| Men | 53 | 69.7% |
| Women | 23 | 30.3% |
| **Diabetes mellitus** |  |  |
| Yes | 27 | 35.5% |
| **High blood pressure** |  |  |
| Yes | 26 | 34.2% |
| **Dyslipidemia** |  |  |
| Yes | 19 | 25% |
| **Smoking habit** |  |  |
| Active smoker | 15 | 19.7% |
| Former smoker | 28 | 36.8% |
| Never smoker | 33 | 43.4% |
| **Obesity** |  |  |
| Underweight | 1 | 1.3% |
| Normal weight | 25 | 32.9% |
| Overweight | 27 | 35.5% |
| Obesity | 21 | 27.6% |
| Morbid obesity | 2 | 2.6% |
| **Civil status** |  |  |
| Single | 13 | 17.1% |
| In a couple/ married | 51 | 67.1% |
| Separated/ divorced | 11 | 14.5% |
| Widower | 1 | 14.5% |
| **Academic level** |  |  |
| Primary | 16 | 21.1% |
| Secondary | 18 | 23.7% |
| Baccalaureate | 13 | 17.1% |
| Vocational training | 16 | 21.1% |
| University | 13 | 17.1% |
| **Employment situation** |  |  |
| Not working/ unemployed | 25 | 32.9% |
| Active | 21 | 27.6% |
| Retiree/ pensioner | 30 | 39.5% |
| **Religion** |  |  |
| Not religious | 27 | 35.5% |
| Practicing Catholic | 32 | 42.1% |
| Non-practicing Catholic | 12 | 15.8% |
| Islamic | 1 | 1.3% |
| Other religions | 4 | 5.3% |
