# Supplementary material for: First-year emotional health after liver transplantation: a prospective cohort study
Source: Front Psychol. 2026 May 19;17:1815413. doi: 10.3389/fpsyg.2026.1815413 (PMC13226472; doi:10.3389/fpsyg.2026.1815413)
Supplement: Supplementary file 2 [file Table_2.docx]

**Supplementary Table 2: Pairwise Comparisons of TxEQ-Spanish Dimensions Over Time**

This supplementary table presents pairwise comparisons of TxEQ-Spanish dimensions across three timepoints (1-, 6-, and 12-months post-transplantation). The comparisons include mean differences, standard errors, Bonferroni-adjusted p-values, and 95% confidence intervals.

| **Dimension** | **Comparison** | **Mean Difference (I-J)** | **Standard Error** | **p-value (Bonferroni)** | **95% CI Lower** | **95% CI Upper** |
| --- | --- | --- | --- | --- | --- | --- |
| Worry | Month 1 vs Month 6 | 0.23 | 0.08 | **0.016** | 0.034 | 0.427 |
| Worry | Month 1 vs Month 12 | 0.135 | 0.09 | 0.423 | -0.087 | 0.357 |
| Worry | Month 6 vs Month 12 | -0.096 | 0.088 | 0.85 | -0.313 | 0.121 |
| Guilt | Month 1 vs Month 6 | -0.011 | 0.074 | 1.0 | -0.192 | 0.17 |
| Guilt | Month 1 vs Month 12 | -0.022 | 0.072 | 1.0 | -0.2 | 0.156 |
| Guilt | Month 6 vs Month 12 | -0.011 | 0.072 | 1.0 | -0.188 | 0.166 |
| Disclosure | Month 1 vs Month 6 | -0.137 | 0.104 | 0.57 | -0.392 | 0.117 |
| Disclosure | Month 1 vs Month 12 | -0.083 | 0.118 | 1.0 | -0.372 | 0.205 |
| Disclosure | Month 6 vs Month 12 | 0.054 | 0.082 | 1.0 | -0.148 | 0.256 |
| Responsibility | Month 1 vs Month 6 | 0.096 | 0.094 | 0.934 | -0.135 | 0.326 |
| Responsibility | Month 1 vs Month 12 | 0.243 | 0.085 | **0.018** | 0.033 | 0.452 |
| Responsibility | Month 6 vs Month 12 | 0.147 | 0.095 | 0.381 | -0.087 | 0.381 |
| Adherence | Month 1 vs Month 6 | -0.018 | 0.068 | 1.0 | -0.184 | 0.149 |
| Adherence | Month 1 vs Month 12 | 0.015 | 0.052 | 1.0 | -0.113 | 0.142 |
| Adherence | Month 6 vs Month 12 | 0.032 | 0.066 | 1.0 | -0.13 | 0.195 |

TxEQ-Spanish: Transplant Effects Questionnaire Spanish

Note: Pairwise comparisons were adjusted using the Bonferroni method to control for Type I error. The significance values reflect this adjustment.
